# Using rice bran as a press aid during the cold‐pressing of sesame seeds improved the extraction yield and quality of the resultant oil

**DOI:** 10.1002/fsn3.4359

**Published:** 2024-08-11

**Authors:** Isa Fathollahy, Behnam Ghaffari

**Affiliations:** ^1^ Department of Food Science and Technology, Mamaghan branch Islamic Azad University Mamaghan Iran

**Keywords:** by‐products, cold‐press, oil quality, press aid, sesame, γ‐Oryzanol

## Abstract

Simple, cost‐effective, and practical techniques can enhance the shelf life of cold‐pressed oils. This study evaluated sesame seed (SS) cold‐pressed oil with various percentages of rice bran (RB) as press aid, including 0% (control or TSSO), 1% (T1), 2.5% (T2.5), 5% (T5), 7.5% (T7.5), and 10% (T10) w/w. The results demonstrated that adding RB considerably boosted extraction yields (*p* < .05), with 5% RB constituting the ideal combination. Adding 7.5% and 10% RB to the SS reduced free fatty acid (FFA) levels compared to the control sample. The oxidative stability index (OSI) and peroxide value (PV), however, significantly increased at combination ratios ranging from 2.5% to 10% (*p* < .05). Rice bran oil (TRBO) had the greatest OSI of all the studied oils, with 18.99 h, while the extracted oils showed an increase in OSI as RB contents rose. Total phenolic compounds (TPC), tocopherols, γ‐oryzanol content, and total antioxidant activity (TAA) increased directly with the RB level. Despite the addition of RB altering the fatty acids concentration, linoleic and oleic acids continued to be the predominant fatty acids in TSSO, TRBO, and other extracted oil samples. Increasing the combination ratio increased the palmitic, palmitoleic, oleic, and linolenic acid content and decreased the stearic and linoleic acid content. In summary, the study demonstrated that the simultaneous cold‐pressing of oil‐bearing seeds and agro‐industrial by‐products is a potentially advantageous technique to increase the extraction yield, qualitative attributes, and shelf life of the extracted oils without the addition of synthetic antioxidants.

## Graphical abstract

Simple, cost‐effective, and practical techniques can enhance the shelf life of cold‐pressed oils. This study evaluated sesame seed (SS) cold‐pressed oil with various percentages of rice bran (RB) as press aid, including 0%, 1%, 2.5%, 5%, 7.5%, and 10% w/w. In summary, the study demonstrated that the simultaneous cold‐pressing of oil‐bearing seeds and agro‐industrial by‐products is a potentially beneficial technique to improve the extraction yield, quality attributes, and stability of the extracted oil without using synthetic antioxidants.

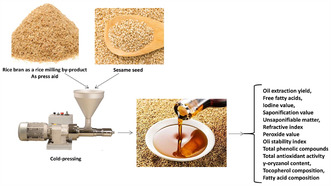

## 1 INTRODUCTION

The proper utilization of food and agro‐industrial processing by‐products and waste can offer benefits in economic, industrial, health, nutritional, and environmental aspects (Torres‐León et al., 2018). RB is an important by‐product generated during the milling and polishing of brown rice into white rice with 12%–18% oil. Depending on rice variety, pretreatments, milling condition, and type of milling system, about 8%–12% of the whole paddy weights are converted into bran (Tan et al., 2023). Due to the presence of bioactive ingredients, antioxidants, and healthy compounds (γ‐oryzanol, tocopherols, and tocotrienols) in RB, it is nutritious (Aparecida et al., 2012). In summary, utilizing by‐products of food and agro‐industrial processing can offer numerous benefits and can provide valuable nutrients and bioactive compounds that are usually wasted. Moongngarm et al. (2012) reported the proximate chemical composition of RB (%dry weight) at 8% degree of milling as follows: carbohydrate (40.63–45.06), fat (15.85–18.8), protein (12.07–13. 66), fiber (11.77–12.68), phenolic compound (1.57–6.65 mg GAE/g), phytic acid (35–50.68 mg/g), γ‐oryzanol (1.52–9.12 mg/g), α‐tocopherol (41–46.12 μg/g), and γ‐tocopherol (25–40.94 μg/g). Rice bran oil stands out from other edible vegetable oils due to its distinctive blend of saturated (SFA: 20% palmitic acid), monounsaturated (MUFA: 42% oleic acid), and polyunsaturated fatty acids (PUFA: 32% linoleic acid), which are balanced in an average ratio of 0.6:1.1:1.0 (Lai et al., 2019). SS, with a high oil content of about 50%, is commonly used for cold pressing. Tocopherols, sesamol, sesamolin, sesamin, inorganic elements, and PUFAs as health‐promoting compounds are present in SS oil. In many Asian countries, SS oil is a sought‐after flavor enhancer due to its pleasant taste and aroma. Furthermore, SS is utilized in producing various products, including cosmetics, pharmaceuticals, margarines, and shortenings (Imran et al., 2020). In summary, SS oil is a versatile ingredient that offers multiple health benefits and is used in diverse applications.

The fundamental issue with cold‐pressed oils is their stability because changes in their organoleptic properties during storage may impair their eating acceptance. Using a range of available methods, such as employing natural or synthetic antioxidants, cold‐pressing, blending oils, extracting oils from mixed oil seeds, adding natural plants, modifying oil manufacturing technology, and pre‐extraction treatments of oil seeds, improvement in quality and stability of oils can be achieved (Dezashibi et al., 2022). According to the findings of Karimpour and Azadmard‐Damirchi (2021), pressing rapeseed with olive leaves resulted in an oil that is similar to virgin olive oil in terms of phenolic content and has a higher concentration of omega‐3 linolenic fatty acids than olive oil. Rapeseed oil is also rich in phenolic compounds, and it may be unveiled as a fresh, health‐improving product. During the cold‐pressing of the black cumin seed and rosemary leaf mixture, the amount of essential oil in the obtained oil increased (from 0.1% to 0.77%) as the proportion of rosemary leaves increased. With the inclusion of rosemary leaves in the composition, the peroxide value of extracted oils also fell from 20 to 8.8 (meq O_2_/kg oil), and they showed greater oxidation stability over time (Afkhami Sarai et al., 2021). Dehghan‐Manshadi et al. (2020) directed a study to assess the impact of infrared‐assisted steam blanching and different extraction techniques, namely ultrasound‐assisted extraction, cold‐press extraction (CPE), and solvent extraction (SE), on the quality of flaxseed oil. In place of conventional roasting, subjecting Nigella sativa L. seeds to microwave pre‐treatment (at a frequency of 2450 MHz for 1–3.5 min) before hydrolytic pressing can result in increased oxidative stability of the extracted oil, as well as further inactivation of lipase, according to a study by Mazaheri et al. (2019a). Incorporating green tea extract into docosahexaenoic acid (DHA)‐rich oil decreased peroxides and secondary oxidation products levels, leading to improved oxidative stability, as demonstrated by Nain et al. (2021). Pomegranate seed oil quality and stability were both improved using pomegranate peel extract alone or in combination with butylated hydroxytoluene (BHT), as noted by Drinić et al. (2020). According to Mazaheri et al. (2019b), the cold‐press co‐extraction method increased the oxidative stability of the black cumin and sunflower seed oil mixture from 6.78 to 9.69 h. Dezashibi et al. (2022) found that simultaneous cold‐pressing of pomegranate seed oil and green tea resulted in an improved oil yield, total phenol content, and oxidative stability. The addition of natural plants (1 g/100 g of garlic or chili pepper) into cold‐pressed black cumin, flax, and hemp seed oil was discovered to have antioxidant capabilities because they effectively inhibited the process of oxidative changes that occurred while the oils were being stored. The induction time was also prolonged by the additions (Krajewska & Kachel, 2022). Cooking oil derived from combining SS oil with RB oil exhibits significant therapeutic effects, such as lipid‐lowering and antihypertensive properties, as reported by Devarajan et al. (2016).

Consumers are increasingly drawn to cold‐pressing as an environmentally friendly, chemical‐free, cost‐effective, and faster method of oil extraction that yields oil with superior nutritional qualities and unique flavor and aroma, according to Fathollahi et al. (2021). While cold pressing offers numerous benefits, its main drawback is its relatively low extraction efficiency, which leaves 8%–14% of the total oil in the cake, as noted by Punia et al. (2021).

The effectiveness of antioxidants is particularly crucial for PUFA‐rich cold‐pressed oils since PUFAs are very susceptible to oxidation, which increases the danger of oil deterioration and, ultimately, negative health impacts when they are consumed. Using simple and low‐cost industrial methods to benefit from the comprehensive advantages of agricultural by‐products, instead of using them as animal feed, is much more favorable and economical for the food industry. Oils are typically stabilized by using extracts and essential oils of plants with antioxidant properties, but this requires the extraction of the extract and essential oil, which is time‐consuming and expensive. A new technique involves using the plant alongside the oil seed during oil extraction. Therefore, from a nutritional and commercial perspective, this effort was done to evaluate the impact of combination of rice bran as a press aid and low‐cost resource with sesame seed on extraction yield and quality characteristics of cold‐pressed sesame oil.

## 2 MATERIALS AND METHODS

### 2.1 Materials

Freshly milled RB samples (with an 8% degree of milling) were collected directly from a local milling factory in Mianeh. White SS were prepared at a local grocery market. Both RB and SS samples were made airtight in black polyethylene bags and stored at 4°C. Gallic acid, tocopherol, fatty acid methyl esters (FAMEs) standard, and 1,1‐diphenyl‐2‐picrylhydrazyl (DPPH) were purchased from Sigma Chemical Co. Acetonitrile, dichloromethane, acetic acid, and Folin–Ciocalteau reagent were prepared from E. Merck Co. The remaining reagents were of analytic quality.

### 2.2 Sample preparation

Mixtures of RB with SS in proportions of 0 (control), 1%, 2.5%, 5%, 7.5%, and 10% (w/w) and with adjusted humidity (8% wet base) were cold‐pressed (Iran cold pressing, Kerman, Iran; single head, 0.75 kW power, 10 kg seed/h capacity, 10 rpm screw rotation speed). The extracted cold‐pressed oils with a temperature lower than 40°C were instantly centrifuged (9000 rpm, 15 min) (Universal, PIT 320) and stored at a nitrogen atmosphere in amber‐colored glasses and in the fridge before analyses. Also, RB oil was cold‐pressed using the method mentioned above. The extraction yield was obtained using the ratio of the obtained crude oil to the pressed raw material (% w/w).

### 2.3 Physicochemical characteristics

The American Oil Chemists' Society (AOCS) methods of Ca 2d‐25 (moisture), Ba 4d‐90 (protein), Ba 5a‐49 (ash), Am 2‐93 (fat), and Ba 6a‐05 (crude fiber) were used to measure the proximate composition of sesame seeds and rice bran. The carbohydrate was calculated by the difference method.

The AOCS' official methods (1997) were used to determine the physicochemical characteristics of oils: Ca 5a‐40 FFA, Cd 8–53 for PV, Cd 1C‐85 for iodine value (IV), Cd 3–25 for saponification value (SV), Ca 6a‐40 for unsaponifiable matters (USM), and Cc 7–25 for refractive index (RI).

Using AOCS' Cd 12b‐92 method, the OSI of the oil samples (2.5 g) was determined by a rancimat instrument (110°C with an airflow rate of 9 Lh^−1^) (Model 743; Metrohm Ltd).

### 2.4 Fatty acid composition

After the preparation of FAMEs according to AOCS' method Ce 2–66, gas chromatography (Shimadzu, 2030 Nexis) using a flame ionization detector (FID) (Agilent Technologies) and a DM‐2330 capillary column (60 m × 0.25 mm i.d., 0.2 μm film thickness; Dikma Technologies Inc.) was used to FAMEs quantification as follows: initial oven temperature of 60°C for 2 min, increase to 200°C (10°C/min) and hold for 10 min, increase to 240°C (5°C/min) and hold for 5 min; 1 μL injection volume, injector split ratio of 1:60; 2 mL/min flow rate of carrier gas. Hydrogen was used as the carrier gas, and the injector and detector were set at 250°C and 260°C, respectively. Retention times comparison with peaks of the mixture of standard fatty acid methyl esters was used for fatty acid peaks identification.

### 2.5 γ‐Oryzanol content

According to the Mariod et al.'s (2014) method, γ‐oryzanol content was determined using reversed phase high performance liquid chromatography (RP‐HPLC) using a Shimadzu (model L‐6200A) HPLC system equipped with a C18 column and a diode‐array ultraviolet–visible detector (Hewlett‐Packard) at 325 nm. Methanol, acetic acid, acetonitrile, and dichloromethane were included in the mobile phase (50:44:3:3 by volume), with a flow rate of 1.4 mL/min and a 20 μL injection volume. Peak area comparisons with those of standards were used to calculate the γ‐oryzanol content.

### 2.6 Tocopherol composition

Following the AOCS Ce 8–89 method, 2 g of seed oil was diluted in a 25 mL volumetric flask, and 20 μL of the diluted sample was injected into an HPLC (Young Lin Acme 9000, YL Instruments Co., Ltd.,) equipped with a normal‐phase silica HPLC column (150 mm × 4.6 mm × 0.5 μm, Sil‐M51001546, HECTOR‐M, RStech) and a fluorescence detector (Jasco FP‐4025, JASCO International Co., Ltd.; λ excitation = 290 nm, and λ emission = 330 nm) to analyze the tocopherol concentrations. The isocratic mobile phase comprised isopropanol:hexane (0.5:99.5 v/v) at a flow rate of 0.5 mL/min at 30°C. The retention times and peak area values were compared with those of a mixed tocopherol standard (in a hexane solution (2 μg/mL)) to determine the tocopherol concentrations.

### 2.7 TPC analysis

To quantify the TPC of the oil samples, Folin–Ciocalteu's reagent method described in detail by Karakaya and Şimşek (2011) was used using a UV–Vis spectrophotometer (Pharmacia Biotech Ltd.) at 725 nm. The results were expressed as gallic acid equivalents (GAE) (mg GAE/100 g oil) (*R* ^2^ = .992).

### 2.8 TAA analysis

In accordance with the method described by Karakaya and Şimşek (2011), a mixture comprising 50 μL of oil and 950 μL of 0.030 mg/mL methanol solution of DPPH radical was vigorously shaken and allowed to stand for 5 minutes in the dark. Subsequently, the absorbance of the mixture was measured against methanol (as a blank) at 515 nm, and the DPPH scavenging activity was expressed as the inhibition of free radical DPPH.

$$\text{Inhibition}\;\left(\%\right)=\left(1-\frac{A_{\text{sample}}}{A_{\text{blank}}}\right)\times 100$$

where A_sample_: absorbance of the sample, A_blank_: absorbance of the DPPH. IC50 (as sample concentrations providing 50% inhibition of DPPH) was calculated from the graph plotted between % inhibitions against sample concentrations.

### 2.9 Statistical analysis

All the treatments were done in triplicate, and the data were presented as mean ± standard deviation. Using Minitab version 21.2 (Minitab), ANOVA and Tukey's mean comparing test were used for the mean comparison at the significance level of *p* < .05.

## 3 RESULTS AND DISCUSSION

### 3.1 SS and RB composition

According to composition analysis, SS comprises 15.86% carbohydrate, 4.21% moisture, 52.36% crude oil, 19.71% crude protein, 3.3% crude fiber, and 4.54% ash (Table 1). The proximate compositions of SS were found to be similar to those reported in other geographical regions (Gharby et al., 2017). Despite the unique combination of crude proteins, the economic and commercial importance of SS is mostly derived from their high oil content.

**TABLE 1:** Proximate composition of sesame seed and rice bran.

	Moisture	Protein	Fiber	Oil	Ash	Carbohydrate
Sesame seed	4.21 ± 0.07	19.71 ± 0.56	3.30 ± 0.25	52.36 ± 1.07	4.54 ± 0.27	15.86 ± 0.85
Rice bran	10.06 ± 0.21	13.20 ± 0.29	21.35 ± 0.93	20.82 ± 0.32	8.83 ± 0.44	25.71 ± 0.88

The proximate composition of RB samples was in the order of moisture (10.06%), crude oil (20.82%), crude protein (13.20%), crude fiber (21.35%), ash (8.83%), and 25.71% carbohydrate (Table 1). Sirikul et al. (2009) reported 9.85%–9.99% moisture, 14.92%–15.17% crude oil, 11.01%–11.77% crude protein, 16.47%–19.94% crude fiber, 13.28%–16.63% ash, and 26.89%–34.08% carbohydrate as proximate components of conventional and organic RB. Based on the factors influencing the chemical composition (genetics, environment, cultivation practices, post‐harvest practices, milling process), the approximate composition of RB varies from one research to another (Kalpanadevi et al., 2018; Moongngarm et al., 2012; Rosniyana et al., 2007).

### 3.2 Oil yield and physicochemical characteristics

Oil yield and physicochemical characteristics of oil samples obtained from different combination ratios of RB and SS are listed in Table 2. The oil yield of TSSO and TRBO was 45.14% and 5.65%, respectively, and incorporation of RB at a combination ratio of 2.5, 5, 7.5, and 10% significantly (*p* < .05) increased extraction yield compared with TSSO (control). The highest oil yield was related to the T5 treatment. Process parameters, such as feeding rate, temperature, and rotation speed, significantly influence oil yield in cold‐pressing methods. These parameters, along with the characteristics of the raw material (dehulling, humidity, oil content, and type of raw material), significantly influence the yield of CPE. It seems that the presence of RB causes more crushing of the SS cell walls and better release of oil due to pressing. In higher proportions of RB, due to its low oil content, it likely absorbs SS oil, reduces oil yield, or works as a barrier to prevent the oil leaking from the ground seed texture. TSSO oil yields (45.14 ± 0.17) in this study were found within the range reported for CPE yields of SS grown in different geographical origins (36%–56%) (Gharby et al., 2017). Dezashibi et al. (2022) observed that the extraction yield of pomegranate seed oil was not significantly affected by the integration of green tea leaves (GTL) (2.5%–5%) (*P* > .05). However, the extraction yield was considerably affected (*p* < .05) by the incorporation of more than 7.5% GTL. Oil extraction using pressurized propane from SS + olive leaves and chia seeds + olive leaves produced lower yields (20 and 17.89%, respectively) than oil extraction using only seeds. The physical obstacles of the shredded leaves preferring preferred paths inside the extraction vessel, the low oil content in the composition of olive leaves, and the low solubility of olive leaf components in propane were used to explain this behavior (Jaski et al., 2023). Oil yields using the CPE method for white (Khao Dawk Mali 105), red (Red Jasmine), and black (Hom‐nin) RB were 5.45%, 6.22%, and 3.22%, respectively, which provided a lower oil yield than SE and supercritical CO_2_ extraction (SC‐CO_2_) methods for about 75% and 65%, respectively (Mingyai et al., 2017). Different extraction methods, including hexane, hot‐pressing, cold‐pressing, and supercritical fluid extraction, recovered 10.92%–12.89%, 5.59%–7.01%, 4.04%–5.80% and 7.06%–9.60%, respectively, of the oil of different rice cultivars (Chiang Mai Black rice, Mali Red rice, and Suphanburi‐1 Brown rice) bran (Pengkumsri et al., 2015). These results showed that the oil yield of RB depends on extraction methods and rice cultivars. The higher content of FFA in TRBO (2.2 ± 0.04%) made its combination with SS, especially in higher proportions, increase the FFA content in the extracted oil (Table 2). The higher amount of FFA in TRBO can be due to the increased activity of lipase after the milling process (Mingyai et al., 2017). A higher amount of FFA is a sign of improper storage of raw materials and low quality of extracted oil (Drinić et al., 2020). The PV and FFA content of all oil samples in this study was lower than the recommended value for cold‐pressed oils by Codex (up to 15 meq O2/kg oil and 4.0 mg KOH/g oil, respectively). Among the extracted oil samples, the lowest and highest PV were related to TRBO and TSSO, respectively, and by increasing the combination ratio from 2.5% to 10%, PV decreased (*p* < .05). Although the incorporation of RB increased the FFA content of extracted oils, the presence of γ‐Oryzanol, tocopherols, and more phenolic compounds (Table 3), which have been released from RB into the extracted oil, can reduce the oxidation rate due to their antioxidant activity (Costa et al., 2019; Maqsood et al., 2014). Delaying the onset of oxidation and preventing its propagation in oils prone to oxidation can be achieved by adding an effective and sufficient, but not excessive, amount of polyphenol rich sources. However, the higher the polyphenol content, the higher the peroxyl radical production, which increases oxidation (Dezashibi et al., 2022; Mazaheri et al., 2019b). Kostadinović Veličkovska et al. (2015) reported 0.2 ± 0.01% FFA and 4.8 ± 0.1 (meq O2/kg of oil) PV for cold‐pressed SS oil from Macedonia.

**TABLE 2:** Physicochemical characteristics of oil samples obtained from different combination ratios of RB and SS.

Treatment	Oil yield (%)	FFA (%)	PV (meq O_2_/kg oil)	IV (gI/100 g oil)	SV (mg KOH/g oil)	USM (%)	RI (25°C)	OSI (h)
TRBO	5.65 ± 0.13 e	2.20 ± 0.04 a	2.90 ± 0.05 c	109.95 ± 0.15 b	186.45 ± 0.72 a	3.42 ± 0.10 a	1.4565 ± 0.0005 c	18.99 ± 0.24 a
TSSO	45.14 ± 0.17 d	0.69 ± 0.02 de	4.93 ± 0.45 a	110.22 ± 0.30 ab	185.55 ± 0.68 a	1.35 ± 0.02 d	1.4706 ± 0.0005 a	9.71 ± 0.4 f
T1	45.22 ± 0.15 d	0.66 ± 0.01 e	4.75 ± 0.07 a	109.83 ± 0.12 b	186.03 ± 0.74 a	1.37 ± 0.03 d	1.4708 ± 0.0007 a	10.17 ± 0.02ef
T2.5	47.85 ± 0.13 b	0.71 ± 0.01 cde	4.09 ± 0.12 b	110.06 ± 0.18 b	185.21 ± 0.36 a	1.40 ± 0.02 d	1.4708 ± 0.0007 a	10.40 ± 0.05 e
T5	51.71 ± 0.17 a	0.72 ± 0.00 cd	3.32 ± 0.19 c	110.83 ± 0.34 a	184.97 ± 0.13 a	1.66 ± 0.06 c	1.4696 ± 0.0002 a	11.35 ± 0.11 d
T7.5	47.65 ± 0.11 b	0.76 ± 0.01 bc	3.12 ± 0.09 c	110.78 ± 0.22 a	185.75 ± 0.46 a	2.15 ± 0.16 b	1.4691 ± 0.0007 a	12.95 ± 0.2 c
T10	47.14 ± 0.13 c	0.80 ± 0.01 b	3.04 ± 0.06 c	110.49 ± 0.26 ab	185.91 ± 0.37 a	2.35 ± 0.11 b	1.4668 ± 0.0007 b	15.51 ± 0.19 b

*Note*: The columns with dissimilar letters (a, b, c, d, e, f) have significant differences in values (*p* < .05). Each mean ± SD represents three replications.

Abbreviations: FFA, free fatty acid; IV, iodine value; OSI: oil stability index; PV, peroxide value; RB, rice bran; RI, refractive index; SS, sesame seed; SV, saponification value; T1, T2.5, T5, T7.5, and T10 represent: 1%, 2.5%, 5%, 7.5%, and 10% combination ratio of RB, respectively; TSSO, pure rice bran oil; TSSO, pure sesame seed oil;

USM, unsaponifiable matter.

**TABLE 3:** TPC, TAA, γ‐oryzanol content, and tocopherol composition of oil samples obtained from different combination ratios of RB with SS.

Treatment	TPC (mg GAE/100 g oil)	TAA (IC50, mg/ml)	γ‐Oryzanol (mg/g)	α‐Tocopherol (mg/g)	β‐Tocopherol (mg/g)	γ‐Tocopherol (mg/g)	δ‐Tocopherol (mg/g)
TRBO	18.83 ± 0.367 a	0.903 ± 0.025 a	34.803 ± 1.296 a	0.206 ± 0.025 a	0.043 ± 0.011 a	0.4 ± 0.060 c	0.026 ± 0.005 b
TSSO	10.527 ± 0.398 f	0.73 ± 0.03 e	ND	0.009 ± 0.000 b	ND	0.405 ± 0.001 c	0.032 ± 0.000 ab
T1	10.763 ± 0.070 ef	0.726 ± 0.015 e	ND	0.009 ± 0.000 b	ND	0.407 ± 0.000 c	0.032 ± 0.000 ab
T2.5	11.386 ± 0.142 e	0.76 ± 0.01 de	0.31 ± 0.04 c	0.009 ± 0.000 b	ND	0.410 ± 0.000 bc	0.032 ± 0.000 ab
T5	12.256 ± 0.170 d	0.806 ± 0.015 cd	1.163 ± 0.070 bc	0.010 ± 0.000 b	0.011 ± 0.000 a	0.431 ± 0.000 abc	0.032 ± 0.000 ab
T7.5	12.98 ± 0.117 c	0.833 ± 0.015 bc	1.62 ± 0.065 bc	0.011 ± 0.000 b	0.060 ± 0.078 a	0.473 ± 0.000 ab	0.032 ± 0.000 a
T10	15.543 ± 0.244 b	0.873 ± 0.015 ab	2.136 ± 0.041 b	0.015 ± 0.000 b	0.020 ± 0.000 a	0.491 ± 0.001 a	0.035 ± 0.000 a

*Note*: The columns with dissimilar letters (a, b, c, d, e, f) have significant differences in values (*p* < .05). Each mean ± SD represents three replications.

Abbreviations: IC50: Half‐maximal inhibitory concentration; RB, rice bran; SS, sesame seed; T1, T2.5, T5, T7.5, and T10 represent 1%, 2.5%, 5%, 7.5%, and 10% combination ratio of RB, respectively; TAA, total antioxidant activity; TPC, Total phenolic compounds; TSSO, pure rice bran oil; TSSO, pure sesame seed oil.

Compared to TSSO treatment, increasing the combination ratio of RB did not significantly (*p > .05*) change the IV and SV of the oil samples. Compared to TSSO treatment, increasing the proportion of RB increased the amount of USM and decreased the RI in T10 oil samples (*p* < .05). The limits of USM for SS oil and RB oil were reported as <2 and 2%–5%, respectively (Gopala Krishna et al., 2003). Jalili et al. (2019) reported the amount of USM in the range of 0.65%–1.87% for 18 samples of cold‐pressed sesame oil produced in Iran. Compared to other vegetable oils, oryzanol, tocopherol, squalene, sterol, tocotrienols, and fatty alcohol as USM are present in higher amounts in the RB oil (4.36%), which have healthy and antioxidant activity (Ahmad Nayik et al., 2015; Farhoosh et al., 2013; Sahu et al., 2018). In USM of Tarom and Fajr RB oil, TPC and antioxidant activity (DPPH method) were 35.65 and 44.96 mg GAE/g and 85.63% and 86.80%, respectively. Considering the satisfactory potential of USM as an antioxidant, encapsulated powders of USM have been used as neutral antioxidants in food formulations (Jamshidi et al., 2020). Research results have shown a significant reduction in the antioxidant properties of palm, olive, and sunflower oils due to the removal of USM from these oils (Dhavamani et al., 2014).

### 3.3 OSI and TAA of the oil samples

Increasing the combination ratio of RB with SS significantly (*p* < .05) increased the OSI of the resulting oil from 9.71 h (TSSO) to 15.51 h (T10) (Table 2). As the most important quality and stability index of edible oils, oxidative stability depends on the antioxidant type and level, temperature, oxidation process products, oil impurities, and the composition of fatty acids (mainly unsaturated fatty acids (UFA)) (Symoniuk et al., 2022). This result can be due to the fact that by increasing the mixing ratio of RB, the amount of linoleic acid decreases, while the amount of total phenol, γ‐oryzanol, γ‐tocopherol, and γ‐tocopherol increases (Tables 3 and 4). GLT methanolic extract was combined with RBD (refined, bleached, and deodorized) palm olein (Womeni et al., 2016); sunflower and black cumin seed oils were extracted simultaneously (Mazaheri et al., 2019b); and pomegranate seed oil was extracted simultaneously with GLT (Dezashibi et al., 2022), all of which improved the oxidative stability associated with the presence of high phenolic compounds. Mazaheri et al., 2019a, 2019b suggested that blending the seeds with a high content of bioactive components before pressing is more effective than the case where the oils are first extracted and then mixed. The degree of unsaturation is very important in the sensitivity of fatty acids to oxidation; therefore, linolenic acid (with 3 double bonds) and linoleic acid (with 2 double bonds) compared to oleic acid (with 1 double bond) are oxidized 25 and 12 times more, respectively (Li et al., 2013). According to Lutterodt et al. (2011), comparing the PUFAs of cold‐pressed concord, muscadine, chardonnay, and ruby red grape seed oils with the OSI (26.9, 19.7, 23.4, and 40.0 h, respectively) interestingly suggests that other factors, like the presence of polyphenolics and other natural antioxidants, may also contribute to oil oxidation stability despite the importance of degree of unsaturation. Redondo‐Cuevas et al. (2018) conducted a linear regression correlation analysis to identify the major contributors to the oxidative stability of oils. They found that TPC and SFA had a positive correlation with the induction period, while UFA, PUFA, and total tocopherols had a negative correlation with the induction period. In other words, higher levels of TPC and SFA were associated with greater oxidative stability, whereas higher levels of UFA, PUFA, and total tocopherols were associated with lower oxidative stability.

**TABLE 4:** Fatty acid composition of oil samples obtained from different combination ratios of RB and SS.

Treatment	Palmitic acid C16:0	Palmitoleic acid C16:1	Stearic acid C18:0	Oleic acid C18:1	Linoleic acid C18:2	Linolenic acid C18:3	Arashidic acid C20:0	SFA	UFA	SFA/UFA
TRBO	21.42 ± 0.43 a	0.45 ± 0.02 a	2.43 ± 0.08 e	43.53 ± 0.59 ab	32.48 ± 0.62 e	1.08 ± 0.05 a	0.56 ± 0.01 a	23.85 ± 0.46 a	78.12 ± 1.17 d	3.27 ± 0.06 d
TSSO	9.73 ± 0.11 d	0.13 ± 0.01 d	5.04 ± 0.10 a	40.17 ± 0.42 d	43.23 ± 0.41 a	0.49 ± 0.02 e	0.57 ± 0.01 a	14.77 ± 0.14 b	84.60 ± 0.82 ab	5.72 ± 0.09 ab
T1	9.81 ± 0.02 d	0.14 ± 0.00 cd	5.13 ± 0.02 a	40.65 ± 0.08 cd	43.65 ± 0.06 a	0.5 ± 0.01 e	0.56 ± 0.01 a	14.94 ± 0.03 b	85.51 ± 0.07 a	5.72 ± 0.01 ab
T2.5	9.85 ± 0.025 d	0.15 ± 0.00 cd	5.13 ± 0.02 a	40.82 ± 0.04 cd	43.08 ± 0.10 a	0.52 ± 0.00 de	0.56 ± 0.01 a	14.98 ± 0.04 b	85.14 ± 0.14 ab	5.68 ± 0.02 ab
T5	10.10 ± 0.02 d	0.17 ± 0.01 c	4.75 ± 0.06 b	41.17 ± 0.05 c	41.16 ± 0.08 b	0.57 ± 0.00 d	0.58 ± 0.01 a	14.86 ± 0.08 b	83.66 ± 0.09 b	5.63 ± 0.03 b
T7.5	10.68 ± 0.07 c	0.17 ± 0.01 c	3.37 ± 0.04 d	42.79 ± 0.07 b	37.16 ± 0.05 c	0.71 ± 0.02 c	0.56 ± 0.00 a	14.06 ± 0.03 c	81.41 ± 0.07 c	5.79 ± 0.01 a
T10	11.25 ± 0.06 b	0.21 ± 0.00 b	3.83 ± 0.04 c	43.59 ± 0.04 a	35.29 ± 0.09 d	0.86 ± 0.01 b	0.57 ± 0.01 a	15.08 ± 0.04 b	80.54 ± 0.10 c	5.33 ± 0.01 c

*Note*: The columns with dissimilar letters (a, b, c, d, e) have significant differences in values (*p* < .05). Each mean ± SD represents three replications.

Abbreviations: RB: rice bran; SFA: saturated fatty acid; SS: sesame seed; T1, T2.5, T5, T7.5, and T10 represent 1%, 2.5%, 5%, 7.5%, and 10% combination ratio of rice bran, respectively; TSSO: pure rice bran oil; TSSO: pure sesame seed oil; UFA: unsaturated fatty acid.

In this study, among oil samples, TRBO showed the highest TAA (IC50), and by increasing RB combination ratio, TAA (IC50) of the obtained oil samples increased significantly (*p* < .05). The IC50 value (in the DPPH free radical scavenging method) is an indicator of the sample's antioxidant capacity, with a lower IC50 value indicating a higher antioxidant capacity or free radical scavenging activity. According to Mingyai et al. (2017), there were significant variations in the scavenging capabilities of colored rice bran oil samples, depending on the extraction methods and varieties used. The authors found that RB oil samples extracted from Hom‐nin rice (black rice) using three different methods (CPE, SE, and SC‐CO_2_) had the least inhibitory concentration at 50% when compared to RB oil samples from Khao Dawk Mali 105 (white rice) and Red Jasmine rice (red rice). Additionally, the RB oil sample extracted from Hom‐nin rice using SC‐CO_2_ had the lowest IC50 value among all the samples tested. Overall, the study's findings suggest that RB oil samples derived from colored rice varieties demonstrated superior antioxidant activity compared to those from white rice. Commercial vegetable oils frequently incorporate synthetic antioxidants to enhance their shelf life, allowing them to remain on the market for a more extended period. Jaski et al. (2022) reported that the combined extraction of active compounds from olive leaves and sunflower oil resulted in an induction period increase of approximately 2.7 and 3.7 h for the pressurized propane and propane and soxhlet methods, respectively, compared to sunflower oil alone. Jaski et al. (2022) noted that the efficacy of antioxidants is related to their oil solubility, with lipophilic antioxidants providing better protection against oxidation. Thus, combining vegetable matrices, such as SS and RB, in a single extraction process can facilitate the acquisition of oil‐soluble antioxidants. The discovery of enhanced protection in cold‐pressed co‐extracted oils (such as SS + RB) with RB compounds can explain the substantial increase observed. This technology offers potential for substituting synthetic antioxidants commonly used in RBD oils, utilizing residue from by‐product processing. The results indicate that this process has a high potential for producing oils with improved oxidative stability without the need for synthetic antioxidants post‐extraction. Enhancing the resistance of vegetable oils to degradation is crucial for the food industry, as it increases their shelf life without using synthetic antioxidants. This value addition to the final product is beneficial for the industry without compromising the health of consumers.

### 3.4 TPC, tocopherol, and γ‐oryzanol content of oil samples

TPC is a critical quality parameter that helps determine the shelf life, nutritional value, and sensory properties of oil. Additionally, TPC serves as a potent antioxidant in the oil. Among the treatments, TRBO oil exhibited the highest total phenolic content (18.83 ± 0.367 mg gallic acid/100 g of oil), whereas TSSO oil had the lowest total phenolic content (10.527 ± 0.398 mg GAE/100 g of oil). The oil of T10 treatments had the highest polyphenol content (15.543 ± 0.244 mg GAE/100 g of oil), which could be attributed to the increased proportion of RB in the mixture. With the increase in the RB combination ratio to 10%, the content of γ‐oryzanol, and γ‐tocopherol also increased to 2.136 (mg/g) and 0.491 (mg/g), respectively (*p* < .05). Due to its low oil content, RB residue retains a significant portion of the oil‐soluble bioactive compounds after pressing. The higher oil content of SS allows it to act as a solvent and carrier for the RB bioactive compounds. The effectiveness of tocopherols as antioxidants depends on their isomers and concentrations. δ‐tocopherol has the highest free radical scavenging activity, followed by γ, β, and α‐tocopherol (Ananth et al., 2019). However, α‐tocopherol has been identified as the most effective scavenger (Yanishlieva et al., 2002). In the present study, the concentration of γ‐tocopherol was high in all of the oil samples.

Kostadinović Veličkovska et al. (2015) reported TPC of 133.42 ± 22.51 (mg GAE/kg oil) and γ‐tocopherol and δ‐tocopherol content of 51.6 ± 0.1 and 0.5 ± 0.0 (mg/100 g oil), respectively, for cold‐pressed SS oil from Macedonia, which was higher than our results. The TPC, total α‐tocopherol, and γ‐oryzanol content in cold‐pressed white, red, and black rice bran oil samples were found to be in the ranges of 6.84–9.26 mg GAE/g oil, 106.90–165.89 mg/g oil, and 0.96–1.84 mg/g oil, respectively, according to Mingyai et al. (2017). Mingyai et al. (2017) reported that pigmented RB oil had higher concentrations of antioxidants, including TPC, γ‐oryzanol, and α‐tocopherol, compared to non‐pigmented RB oil. In terms of extraction method, the solvent extraction method resulted in the highest content of γ‐oryzanol, followed by the SC‐CO_2_ and CPE methods, respectively. This is due to the fact that γ‐oryzanol is soluble in organic solvents. Mingyai et al. (2017) found that the RB oil sample extracted by the CPE method had the highest α‐tocopherol content, followed by the SE and SC‐CO_2_ methods, respectively. These results suggest that each extraction method may be more suitable for extracting a specific compound than all of the compounds present in the RB oil. According to Dezashibi et al. (2022) and Mazaheri et al. (2019b), the addition of GTL to pomegranate seeds and black cumin seed (BCS) to sunflower seeds leads to an increase in the TPC of extracted oils using cold‐pressing. The TPC content of cold‐pressed pomegranate seed oil with 0–10% incorporated GTL ranged from 2.7 to 12.1 mg GAE/g. Similarly, the TPC content of cold‐pressed sunflower seed oil with 0%–15% w/w incorporated BCS ranged from 27.1–323.9 mg caffeic acid/kg oil. According to Jaski et al. (2022), the incorporation of olive leaf into sunflower grains resulted in a 53.11% increase in α‐tocopherol of the obtained oils using the classic extraction technique (Soxhlet method) and a 35.44% increase using pressurized propane extraction. These findings indicate the positive effect of GTL, BCS, and olive leaf incorporation on the TPC of extracted pomegranate and sunflower oil.

The use of olive leaves in the oil extraction vessel of chia and sesame seeds via pressurized propane produced vegetable oils with higher antioxidant activities than pure chia and sesame oils, which was supported by the low IC50 values. During simultaneous extraction, vegetable oils act as co‐solvents for the extraction of active compounds with antioxidant action and choose high‐affinity compounds, resulting in efficient incorporation of compounds in the finished product and offering a novel alternative approach for industrial expansion in the food industry (Jaski et al., 2023).

### 3.5 Fatty acid composition

The nutritional, technological, functional, and oxidative stability properties of edible oils are influenced by their fatty acid composition. In Table 4, the fatty acid composition of TSSO, TRBO, and oils with different combination ratios of RB is presented. The major fatty acids in TRBO were linoleic (C18:2), oleic (C18:1), palmitic (C16:0), and stearic acids (C18:0), accounting for 43.23%, 40.17%, 9.73%, and 5.04%, respectively. TRBO was specified by high levels of oleic acid (43.53%), followed by linoleic acid (32.48%) and palmitic acid (21.42%) (Table 4). The combination of RB with SS led to an increase in palmitic acid, palmitoleic acid (C16:1), oleic acid, and linolenic acid (C18:3), and a decrease in stearic acid and linoleic acid. Compared to other combination ratios, SFA in 7.5%, UFA in 7.5% and 10%, and SFA/UFA in 10% decreased significantly (*p* < .05). In a study by Kostadinović Veličkovska et al. (2015), the cold‐pressed SS oil from Macedonia showed a similar fatty acid composition to that of the present study. Mingyai et al. (2017) observed that the primary SFA in pigmented RB oil were myristic, palmitic, and stearic acids, while the major UFA were palmitoleic, oleic, linoleic, and linolenic acids. Furthermore, the fatty acid composition of RB oil obtained through varied extraction methods, including CPE, SE, and SC‐CO_2_, was significantly different.

## 4 CONCLUSION

To sum up, the results indicate that the combination of RB with SS has a protective effect on maintaining the stability of SS oil, resulting in improved resistance to oxidation during storage. The best extraction efficiency (51.71%) and OSI (15.51 h) were achieved with a ratio of 5% and 10% of RB and SS, respectively.

Blending prior to pressing improved the TPC, TAA, γ‐Oryzanol, β‐tocopherol, and γ‐tocopherol in the extracted oils. Furthermore, combining RB with SS before pressing increased the oleic acid content and decreased the SFA/UFA ratio. Therefore, the simultaneous extraction of oil from a mixture of agro‐food processing by‐products and oil‐bearing seeds is a recommended method for the industry due to its safety, affordability, and efficiency.

## AUTHOR CONTRIBUTIONS

**Isa Fathollahy:** Conceptualization, methodology, writing and editing, data analysis and validation, interpretation. **Behnam Ghaffari:** Carried out the experiment, Aided in interpreting the results, and writing the manuscript.

## FUNDING INFORMATION

None.

## CONFLICT OF INTEREST STATEMENT

The authors declare that there is no conflict of interest in publishing the findings.

### DATA AVALABILITY STATEMENT

The data that support the findings of this study are available from the corresponding author upon reasonable request.

## ETHICS STATEMENT

This research does not involve human or animal testing.
